# Supplementary material for: In employees’ favour or not?—The impact of virtual office platform on the work-life balances
Source: PLoS One. 2021 Nov 19;16(11):e0260220. doi: 10.1371/journal.pone.0260220 (PMC8604353; doi:10.1371/journal.pone.0260220)
Supplement: S1 Appendix — (DOCX) [file pone.0260220.s001.docx]

**S1 Appendix. Questionnaire.**

**THE IMPACT OF VIRTUAL OFFICE PLATFORM ON THE WORK-LIFE BALANCE**

Dear Sir/Madam;

I’m conducting a research on “The impact of virtual office platform on the work-life balance” as partial fulfillment of Master of Business Administration at the Faculty of Business of the Sri Lanka Institute of Information Technology. I am kindly inviting you to participate in this research study by completing the questionnaire which will approximately take fifteen to twenty minutes to complete. Further, I ensure that the information provided will be treated confidential and will only be used for study purposes.

Thank you for taking the time to assist me in my educational endeavors and your participation is highly appreciated.

**Section A – Demographic Factor**

Please select the most suitable answer for each statement.

1. Age : 20 – 25 years 26 – 30 years 31 – 40 years

41 – 50 years > 51 years

1. Gender : Male Female

1. Education : GCE A/L Diploma Degree

Under Graduate Post Graduate

1. Monthly Income : Below 50,000 50,000 – 100,000

100,000 – 150,000 Above 150,000

1. Civil status : Single Married

5.1 If married, does the spouse do a job?

YES No

5.2 Do you have children?

YES No

1. Do you use a virtual office platform or a traditional office platform to execute your work?

Virtual office platform Traditional office platform

Both platforms

1. Do the virtual office hours longer than your regular working hours?

Yes, most of the time No, Never Yes, rarely

1. Do you work on weekends?

Yes, most of the time No, Never Yes, rarely

1. I spend worth time with my family:

Yes No Some times

**Section B**

Please select (×) the most appropriate term (strongly Disagree to strongly agree) for each question below.

| Strongly Disagree | Agree | Neutral | Disagree | Strongly Agree |
| --- | --- | --- | --- | --- |
| 1 | 2 | 3 | 4 | 5 |

| **Q.No** | **Question** | **1** | **2** | **3** | **4** | **5** |
| --- | --- | --- | --- | --- | --- | --- |
|  | **Working Environment** | **1** | **2** | **3** | **4** | **5** |
| 1. | I have a specific room to work in without any interference from family members | **1** | **2** | **3** | **4** | **5** |
| 2. | If I have a specific room for myself it helps me to finish work within fewer hours | **1** | **2** | **3** | **4** | **5** |
| 3. | Having a good working environment helps me to do my job easily | **1** | **2** | **3** | **4** | **5** |
| 4. | My room is sufficient to carry out my official work | **1** | **2** | **3** | **4** | **5** |
| 5. | The presence of people in the household during the work from home period increases the amount of time spent on the same work which can complete within a few hours | **1** | **2** | **3** | **4** | **5** |
| 6. | If there are more people in the house I can’t concern about my office work | **1** | **2** | **3** | **4** | **5** |
| 7. | The number of people available in the house will affect negatively to the quality of work | **1** | **2** | **3** | **4** | **5** |
| 8. | When virtual working days are increasing, it indirectly affects the free time that leaves to spend with the family members | **1** | **2** | **3** | **4** | **5** |
| 9. | Usually, I do not exceed the normal working hours even during work from the home period | **1** | **2** | **3** | **4** | **5** |
| 10. | If I work more in a virtual office it will affect my personal -life | **1** | **2** | **3** | **4** | **5** |
| 11. | I spend the weekend with my family even I have all facilities to work from home during the weekend |  |  |  |  |  |
| 12. | Virtual work has a huge responsibility than regular work | **1** | **2** | **3** | **4** | **5** |
| 13. | Now I can take more responsibility for my family due to the virtual office platform | **1** | **2** | **3** | **4** | **5** |
| 14. | Work responsibilities are more complex during virtual office platform than traditional office platform. | **1** | **2** | **3** | **4** | **5** |
| 15. | I can adjust any family requirement as I wish due to the virtual work platform | **1** | **2** | **3** | **4** | **5** |
| 16. | Due to the virtual office platform, it saves a lot of time consumed for transportation | **1** | **2** | **3** | **4** | **5** |
| 17. | The amount of time spent with the virtual office platform may have a positive effect on my family life | **1** | **2** | **3** | **4** | **5** |
| 18. | The time spent with the virtual office platform to perform duties is less compare to the traditional office | **1** | **2** | **3** | **4** | **5** |
|  | **Non-Working Environment** |  |  |  |  |  |
| 19. | Gender has an impact on working platforms  (Virtual office and traditional platform) | **1** | **2** | **3** | **4** | **5** |
| 20. | There is an impact on virtual office platform on the work-life balance according to gender | **1** | **2** | **3** | **4** | **5** |
| 21. | The number of children in the family mainly affects the work-life balance | **1** | **2** | **3** | **4** | **5** |
| 22. | Virtual office platform is more suitable for parents which are having children | **1** | **2** | **3** | **4** | **5** |
| 23. | There is an impact of the virtual office on the work-life balance  Work from is mostly suitable for presents who are having child. | **1** | **2** | **3** | **4** | **5** |
|  | **Work life Balance** | **1** | **2** | **3** | **4** | **5** |
| 24. | There is an impact of virtual office on family satisfaction | **1** | **2** | **3** | **4** | **5** |
| 25. | Marital Satisfaction is low due to the virtual office work platform | **1** | **2** | **3** | **4** | **5** |
| 26. | With the virtual office platform, I am satisfied with my life comparing to the traditional office life | **1** | **2** | **3** | **4** | **5** |
| 27. | For me, social undermining can happen if I move to the virtual office platform | **1** | **2** | **3** | **4** | **5** |
| 28. | If I use a virtual platform I can gain more personal growth compared to traditional office | **1** | **2** | **3** | **4** | **5** |
| 29. | There is an impact on depression & distress due to virtual office work | **1** | **2** | **3** | **4** | **5** |
| 30. | There is a relationship between Alcohol consumption and virtual work platform | **1** | **2** | **3** | **4** | **5** |
| 31. | Normally I spend sufficient time enhancing my physical health due to virtual office | **1** | **2** | **3** | **4** | **5** |
| 32. | Virtual office platform may lead to Psychological problems in the future | **1** | **2** | **3** | **4** | **5** |

**THANK YOU**
