# Supplementary material for: In employees’ favour or not?—The impact of virtual office platform on the work-life balances
Source: PLoS One. 2021 Nov 19;16(11):e0260220. doi: 10.1371/journal.pone.0260220 (PMC8604353; doi:10.1371/journal.pone.0260220)
Supplement: S2 Appendix — (DOCX) [file pone.0260220.s002.docx]

**S2 Appendix. Data file.**

| Variable |  | Frequency | Percentage % |
| --- | --- | --- | --- |
| 1.Age | 20-25 Years  26-30 Years  31-40 Years  41-50 Years | 34  106  102  28 | 12.6  39.3  37.8  10.4 |
| 2.Gender | Male  Female | 130  140 | 48.1  51.9 |
| 3.Education | GCE A/L  Diploma  Degree  Under Graduate  Post Graduate | 9  38  139  74  10 | 3.3  14.1  51.5  27.4  3.7 |
| 4.Monthly Income | Below 50,000/=  50,000-100,000/=  100,000 - 150,000/=  Above 150,000/= | 23  169  67  11 | 8.5  62.6  24.8  4.1 |
| 5.Civil status | Single  Married | 117  153 | 43.3  56.7 |
| 5.1. If married, does the spouse do a job? | Yes  No  Not answered | 118  81  71 | 43.7  30.0  26.3 |
| 5.2. If married, Do you have children? | Yes  No  Not answered | 130  69  71 | 48.1  25.6  26.3 |
| 6.Do you use a virtual office platform or traditional office platform or both platforms to execute your work? | Virtual office platform  Both platforms | 107  163 | 39.6  60.4 |
| 7.Do the virtual office hours longer than your regular working hours? | No, Never  Yes, Most of the time  Yes, rarely | 15  125  130 | 5.6  46.3  48.1 |
| 8.Do you work on weekends? | Yes, Most of the time  No, Never  Yes, rarely | 132  13  125 | 48.9  4.8  46.3 |
| 9.I spend worth time with my family: | No  Yes  Some times | 5  225  40 | 1.9  83.3  14.8 |
| 1.I have a specific room to work in without any interference from family members | Strongly Disagree  Disagree  Neutral  Agree  Strongly Agree | 2  12  72  162  22 | .7  4.4  26.7  60.0  8.1 |
| 2.If I have a specific room for myself it helps me to finish work within fewer hours | Neutral  Agree  Disagree  Strongly Agree | 25  182  1  62 | 9.3  67.4  .4  23.0 |
| 3.Having a good working environment helps me to do my job easily | Disagree  Neutral  Agree  Strongly Agree | 2  28  163  77 | .7  10.4  60.4  28.5 |
| 4.My room is sufficient to carry out my official work | Strongly Disagree  Disagree  Neutral  Agree  Strongly Agree | 1  8  64  165  32 | .4  3.0  23.7  61.1  11.9 |
| 5.The presence of people in the household during the work from home period increases the amount of time spent on the same work which can complete within a few hours | Disagree  Neutral | 2  268 | .7  99.3 |
| 6.If there are more people in the house I cannot concern about my office work | Disagree  Neutral | 1  269 | .4  99.6 |
| 7.The number of people available in the house will affect negatively to the quality of work | Disagree  Neutral | 1  269 | .4  99.6 |
| 8.When virtual working days are increasing, it indirectly affects the free time that leaves to spend with the family members | Disagree  Neutral | 3  267 | 1.1  98.9 |
| 9.Usually, I do not exceed the normal working hours even during work from the home period | Strongly Disagree  Disagree  Neutral  Agree  Strongly Agree | 2  7  116  120  25 | .7  2.6  43.0  44.4  9.3 |
| 10.If I work more in a virtual office it will affect my personal -life | Disagree  Neutral | 3  267 | 1.1  98.9 |
| 11.I spend the weekend with my family even I have all facilities to work from home during the weekend | Disagree  Neutral  Agree  Strongly Agree | 8  137  108  17 | 3.0  50.7  40.0  6.3 |
| 12.Virtual work has a huge responsibility than regular work | Disagree  Neutral | 1  269 | .4  99.6 |
| 13.Now I can take more responsibility for my family due to the virtual office platform | Disagree  Neutral  Agree  Strongly Agree | 2  71  156  41 | .7  26.3  57.8  15.2 |
| 14.Work responsibilities are more complex during virtual office platform than traditional office platform. | Disagree  Neutral | 1  269 | .4  99.6 |
| 15.I can adjust any family requirement as I wish due to the virtual work platform | Strongly Disagree  Disagree  Neutral  Agree  Strongly Agree | 1  1  74  163  31 | .4  .4  27.4  60.4  11.5 |
| 16.Due to the virtual office platform, it saves a lot of time consumed for transportation | Strongly Disagree  Neutral  Agree  Strongly Agree | 1  52  175  42 | .4  19.3  64.8  15.6 |
| 17.The amount of time spent with the virtual office platform may have a positive effect on my family life | Disagree  Neutral  Agree  Strongly Agree | 2  63  161  44 | .7  23.3  59.6  16.3 |
| 18.The time spent with the virtual office platform to perform duties is less compare to the traditional office | Strongly Disagree  Disagree  Neutral  Agree  Strongly Agree | 1  6  130  102  31 | .4  2.2  48.1  37.8  11.5 |
| 19.Gender has an impact on working platforms (Virtual office and traditional platform) | Strongly Disagree  Disagree  Neutral  Strongly Agree | 3  11  220  36 | 1.1  4.1  81.5  13.3 |
| 20.There is an impact on virtual office platform on the work-life balance according to gender | Strongly Disagree  Disagree  Neutral  Strongly Agree | 2  1  207  60 | .7  .4  76.7  22.2 |
| 21.The number of children in the family mainly affects the work-life balance | Strongly Disagree  Disagree  Neutral  Strongly Agree | 1  2  211  56 | .4  .7  78.1  20.7 |
| 22.Virtual office platform is most suitable for parents which are having children | Strongly Disagree  Disagree  Neutral  Strongly Agree | 1  2  204  63 | .4  .7  75.6  23.3 |
| 23.There is an impact of the virtual office on the work-life balance | Strongly Disagree  Disagree  Neutral  Strongly Agree | 1  1  221  47 | .4  .4  81.9  17.4 |
| 24.There is an impact of virtual office on family satisfaction | Disagree  Neutral  Strongly Agree | 6  232  32 | 2.2  85.9  11.9 |
| 25.Marital Satisfaction is low due to the virtual office work platform | Strongly Disagree  Disagree  Neutral  Strongly Agree | 3  22  232  13 | 1.1  8.1  85.9  4.8 |
| 26.With the virtual office platform, I am satisfied with my life comparing to the traditional office life | Strongly Disagree  Disagree  Neutral  Agree  Strongly Agree | 3  14  87  148  18 | 1.1  5.2  32.2  54.8  6.7 |
| 27.For me, social undermining can happen if I move to the virtual office platform | Strongly Disagree  Disagree  Neutral  Strongly Agree | 2  14  240  14 | .7  5.2  88.9  5.2 |
| 28.If I use a virtual platform I can gain more personal growth compared to traditional office | Strongly Disagree  Disagree  Neutral  Agree  Strongly Agree | 2  5  111  139  13 | .7  1.9  41.1  51.5  4.8 |
| 29.There is an impact on depression & distress due to virtual office work | Strongly Disagree  Disagree  Neutral  Strongly Agree | 2  2  242  24 | .7  .7  89.6  8.9 |
| 30.There is a relationship between Alcohol consumption and virtual work platform | Strongly Disagree  Disagree  Neutral  Strongly Agree | 10  15  241  4 | 3.7  5.6  89.3  1.5 |
| 31.Normally I spend sufficient time enhancing my physical health due to virtual office | Strongly Disagree  Disagree  Neutral  Agree  Strongly Agree | 3  5  87  160  15 | 1.1  1.9  32.2  59.3  5.6 |
| 32.Virtual office platform may lead to Psychological problems in the future | Strongly Disagree  Disagree  Neutral  Strongly Agree | 1  5  222  42 | .4  1.9  82.2  15.6 |
|  | **Total** | **270** | **100 %** |
