# Supplementary material for: In employees’ favour or not?—The impact of virtual office platform on the work-life balances
Source: PLoS One. 2021 Nov 19;16(11):e0260220. doi: 10.1371/journal.pone.0260220 (PMC8604353; doi:10.1371/journal.pone.0260220)
Supplement: S3 Appendix — (DOCX) [file pone.0260220.s003.docx]

**S3 Appendix. Coefficients Table and Model Summary for Model 03**

COEFFICIENTS TABLE FOR MODEL 03

| **Coefficients^a^** | | | | | | | | | | | |
| --- | --- | --- | --- | --- | --- | --- | --- | --- | --- | --- | --- |
| Model | | Unstandardized Coefficients | | Standardized Coefficients | t | Sig. | 95.0% Confidence Interval for B | | Collinearity Statistics | |  |
|  |  | B | Std. Error | Beta |  |  | Lower Bound | Upper Bound | Tolerance | VIF |  |
| 1 | (Constant) | 2.156 | .106 |  | 20.390 | .000 | 1.948 | 2.364 |  |  |  |
|  | NoN_Working_Environment | .331 | .031 | .547 | 10.689 | .000 | .270 | .392 | 1.000 | 1.000 |  |
| 2 | (Constant) | .699 | .259 |  | 2.698 | .007 | .189 | 1.210 |  |  |  |
|  | NoN_Working_Environment | .243 | .033 | .401 | 7.474 | .000 | .179 | .307 | .801 | 1.249 |  |
|  | Working_Environment | .491 | .081 | .326 | 6.083 | .000 | .332 | .650 | .801 | 1.249 |  |
| 3 | (Constant) | .677 | .257 |  | 2.628 | .009 | .170 | 1.183 |  |  |  |
|  | NoN_Working_Environment | .248 | .032 | .410 | 7.676 | .000 | .185 | .312 | .797 | 1.255 |  |
|  | Working_Environment | .484 | .080 | .322 | 6.042 | .000 | .326 | .642 | .800 | 1.250 |  |
|  | A_1_Age=31-40 Years | .076 | .034 | .108 | 2.257 | .025 | .010 | .143 | .995 | 1.005 |  |
| a. Dependent Variable: Work_life_Balance | | | | | | | | | | | |

MODEL SUMMARY FOR MODEL 03

| **Model Summary** | | | | |
| --- | --- | --- | --- | --- |
| Model | R | R Square | Adjusted R Square | Std. Error of the Estimate |
| 1 | .547^a^ | .299 | .296 | .28879 |
| 2 | .620^b^ | .384 | .380 | .27115 |
| 3 | .629^c^ | .396 | .389 | .26909 |
| a. Predictors: (Constant), NoN_Working_Environment | | | | |

**Operationalization Table**

|  | Variable Name | Indicators | References | Question items |
| --- | --- | --- | --- | --- |
| Independent Variable | Working Environment | - Homeworking space - Number of the person in the house - Working days - Responsibility - Time duration | [1]  [2]  [3]  [4]  [5]  [6]  [7]  [8]  [9]  [10] | 1 to 18 |
|  | Non-working  Environment | - Gender - Number of children | [2]  [11]  [4]  [12]  [13]  [10] | 19 to 23 |
| Dependent Variable |  | - Family satisfaction - Marital Satisfaction - Life Satisfaction - Social undermining - Personal growth - Depression & distress - Daily Alcohol consumption - Physical health - Psychological problems | [14]  [6]  [15]  [7]  [16]  [8]  [9] | 24 to 33 |

**References**

1. Solís Martín S. Telework: conditions that have a positive and negative impact on the work-family conflict. Academia Revista Latinoamericana de Administración. 2016;29(4):435-49. doi: 10.1108/ARLA-10-2015-0289.

2. Calvo-Salguero A, Salinas J, Aguilar-Luzón MC. Gender and work–family conflict: Testing the rational model and the gender role expectations model in the Spanish cultural context. International journal of psychology : Journal international de psychologie. 2011;47:118-32. doi: 10.1080/00207594.2011.595414.

3. Carlson D, Kacmar K, Williams L. Construction and Initial Validation of a Multidimensional Measure of Work–Family Conflict. Journal of Vocational Behavior. 2000;56:249-76. doi: 10.1006/jvbe.1999.1713.

4. Adams G, King L, King D. Relationships of Job and Family Involvement, Family Social Support, and Work-Family Conflict With Job and Life Satisfaction. Journal of Applied Psychology. 1996;81:411-20. doi: 10.1037//0021-9010.81.4.411.

5. Greenhaus J, Beutell N. Source of Conflict Between Work and Family Roles. The Academy of Management Review. 1985;10:76-88. doi: 10.2307/258214.

6. Frone M, Yardley J, Markel K. Developing and Testing an Integrative Model of the Work – Family Interface. Journal of Vocational Behavior. 1997;50:145-67. doi: 10.1006/jvbe.1996.1577.

7. Frone M, Russell M, Cooper M. Antecedents and Outcomes of Work-Family Conflict: Testing a Model of the Work-Family Interface. The Journal of applied psychology. 1992;77:65-78. doi: 10.1037//0021-9010.77.1.65.

8. Rice R, Frone M, McFarlin D. Work-nonwork conflict and perceived quality of life. Journal of Organizational Behavior. 1992;13:155-68. doi: 10.1002/job.4030130205.

9. Kopelman R, Greenhaus J, Connolly T. A model of work, family, and interrole conflict: A construct validation study. Organizational Behavior and Human Performance. 1983;32:198-215. doi: 10.1016/0030-5073(83)90147-2.

10. Hamblin H. Employees' Perspectives on One Dimension of Labour Flexibility: Working at a Distance. Work, Employment and Society. 1995;9(3):473-98. doi: 10.1177/095001709593003.

11. Nakrošienė A, Bučiūnienė I, Goštautaitė B. Working from home: characteristics and outcomes of telework. International Journal of Manpower. 2019;40(1):87-101. doi: 10.1108/IJM-07-2017-0172.

12. Lim V, Teo T. To work or not to work at home: An empirical investigation of factors affecting attitudes towards teleworking. Journal of Managerial Psychology. 2000;15:560-86. doi: 10.1108/02683940010373392.

13. Yap CS, Tng H. Factors associated with attitudes towards telecommuting. Information & Management. 1990;19(4):227-35. doi: <https://doi.org/10.1016/0378-7206(90)90032-D>.

14. Le H, Newman A, Menzies J, Zheng C, Fermelis J. Work–life balance in Asia: A systematic review. Human Resource Management Review. 2020;30(4):100766. doi: <https://doi.org/10.1016/j.hrmr.2020.100766>.

15. Greenhaus J, Bedeian A, Mossholder K. Work experiences, job performance, and feelings of personal and family well-being. Journal of Vocational Behavior. 1987;31:200-15. doi: 10.1016/0001-8791(87)90057-1.

16. Md Sidin S, Sambasivan M, Ismail I. Relationship between work-family conflict and quality of life: An investigation into the role of social support. Journal of Managerial Psychology. 2010 25:58-81. doi: 10.1108/02683941011013876.
