# Supplementary material for: In employees’ favour or not?—The impact of virtual office platform on the work-life balances
Source: PLoS One. 2021 Nov 19;16(11):e0260220. doi: 10.1371/journal.pone.0260220 (PMC8604353; doi:10.1371/journal.pone.0260220)
Supplement: Attachment — Submitted filename: Response to Reviewers.docx [file pone.0260220.s006.docx]

**Point–by–point response to reviewers**

**Comments from Authors:** Please note that page numbers and line numbers refereed in this document is align with the revised manuscript which has track changes.

| **Comments of Reviewers** | **Comments of Authors** |
| --- | --- |
| **Comment 1** |  |
| If the authors have adequately addressed your comments raised in a previous round of review and you feel that this manuscript is now acceptable for publication, you may indicate that here to bypass the “Comments to the Author” section, enter your conflict of interest statement in the “Confidential to Editor” section, and submit your "Accept" recommendation.  Reviewer #1: All comments have been addressed  Reviewer #2: All comments have been addressed | Well noted. Thank you for your comment. |
| **Comment 2** |  |
| Is the manuscript technically sound, and do the data support the conclusions?  Reviewer #1: Yes  Reviewer #2: Partly | Thank you comment has been noted.  . |
| **Comment 3** |  |
| Has the statistical analysis been performed appropriately and rigorously?  Reviewer #1: Yes  Reviewer #2: Yes | Thank you very much for your comment. |
| **Comment 4** |  |
| Have the authors made all data underlying the findings in their manuscript fully available?  The PLOS Data policy requires authors to make all data underlying the findings described in their manuscript fully available without restriction, with rare exception (please refer to the Data Availability Statement in the manuscript PDF file). The data should be provided as part of the manuscript or its supporting information, or deposited to a public repository. For example, in addition to summary statistics, the data points behind means, medians and variance measures should be available. If there are restrictions on publicly sharing data—e.g. participant privacy or use of data from a third party—those must be specified.  Reviewer #1: No  Reviewer #2: No | Thank you comment has been noted.  Data cannot be shared publicly because of data protection. Data are available from the Sri Lanka Institute of Information Technology, SLIIT Business School, Sri Lanka (contact via gazrat4ever@gmail.com or ruwan.j@sliit.lk ) for researchers who meet the criteria for access to confidential data. |
| **Comment 5** |  |
| Is the manuscript presented in an intelligible fashion and written in standard English?  PLOS ONE does not copyedit accepted manuscripts, so the language in submitted articles must be clear, correct, and unambiguous. Any typographical or grammatical errors should be corrected at revision, so please note any specific errors here.  Reviewer #1: Yes  Reviewer #2: Yes | Thank you. Well noted. |
| **6. Review Comments to the Author**  **Reviewer 1** | |
| **Abstract**  Common practice to write in words any number under two digits – “9” as “nine”  **Review sentences for succinct delivery and brevity.**  Line 11 – research gap sentence to precede the objective of the study to improve flow.  Suggestion - Line 17 and 18 does can be combined with the following sentence – E.g. “Findings indicate that it is vital that…….. xx xx so that best use of virtual work environments can be obtained”  **Combine line 14, 15, 16 to form one sentence** | Comment has been noted and this has been corrected in the revised manuscript.  (Page 2 - line 5)  This has been corrected in the revised version.  Thank you. Comment has been noted and this has been corrected in the revised manuscript (Page 2 - line 9-13)  Well noted and this has been corrected in the revised manuscript (Page 2 - line 18-21)  Comment has been noted and this has been corrected in the revised manuscript (Page 2 - line 13-18). |
| **Introduction:**  -Overall comment - Review for succinct presentation of ideas and brevity of language. Synthesize and present ideas in summary to the reader removing all and any unncessary information/words (review for brevity). For E.g. Line 24 – “Crosbie and Moore state that homeworking or working from home can improve the work-life balance”. | Comment has been well noted and this has been corrected in the revised manuscript (Page 2 - line 27-29). |
| **Problem statement:**  Problem statement is repetitive of introduction and includes information re: women and literature that should be provided with in the introduction/review section. Problems statement should remain a very clear statement of the problems without other information. | Thank you for your comment. This has been corrected in the revised manuscript (Page 4 - line 31-34 and Page 5 – line 14-17). |
| **Objective of study:**  This section also provides rationale/justification of study which should be provided separately, prior to objective | Comment is well noted and this has been corrected in the revised manuscript. Rational of the study has been stated before the objective (page 6- line 2-21). |
| **Literature review:**  Overall comment - Review for succinct presentation of ideas and brevity of language. Synthesize and present ideas in summary to the reader removing all and any unncessary information/words (review for brevity) | This has been corrected in the revised version. Revised version has been proofread. Correction has been made including typos, comma correction and brevity. |
| **Methodology:**  The first sentence repeats purpose of study - please remove  Subtopic 'Data' can be removed with passage directly under method.  Page 16, conceptual model diagram is repeated and must be removed.  Hypothesis should logically be provided under the problem statement or within that section, and not under method.  Methodology can include measure development information in it as part of it, and results containing all results including demographics from the study - please revise - confusing to reader  A discussion section is presented before analysis - the use of topics/subtopics are confusing to the reader  Why is reliability analysis of the tool provided after presenting demographic information of the study population? Please be mindful of the flow of the article  Clearly demark 'discussion' following results presentation and discuss all matters related to findings under that subtopic | Thank you for your comment. This has been corrected in the revised manuscript with track changes (Page 15 - line 1).  Comment is well noted. This has been corrected in the revised manuscript with track changes (Page 14 - line 30).  Comment is well noted. As per the suggestion, conceptual model sub section has been removed and manuscript adjusted accordingly.  Thank you for your comment. This has been corrected in the revised manuscript with track changes (Page 5 - line 25-32).  This has been corrected in the revised version. Now methodological section includes the development information (questioner development and the analytical tool). Results section includes the descriptive statistics, reliability and regression results.  Thank you for your comment. This has been corrected in the revised manuscript. Result and discussion sections has been separated.  This has been corrected in the revised version. demographic information has been now presented after the reliability analysis (Page 19 - line 3-19 and page 20 line 1-4).  Well noted. Subtopic has been introduced under discussion section (Page 28 – line 3; Page 28 – line 17-18; Page 30 – line 4-5) |
| **Consluions:**  Insert limitations as separate topic­  Review overall article for logical flow of presentation and language edit for brevity. | Thank you for your comment. This has been corrected in the revised manuscript (Page 33 - line 24-31)  This has been corrected in the revised version. Revised version has been proofread. Correction has been made including typos, comma correction and brevity. |
